# Insights into the Structure and Function of Ciliary and Flagellar Doublet Microtubules: TEKTINS, Ca^2+^-BINDING PROTEINS, AND STABLE PROTOFILAMENTS*[Fn FN3]^♦^

**DOI:** 10.1074/jbc.M114.568949

**Published:** 2014-05-02

**Authors:** Richard Linck, Xiaofeng Fu, Jianfeng Lin, Christna Ouch, Alexandra Schefter, Walter Steffen, Peter Warren, Daniela Nicastro

**Affiliations:** From the ‡ Department of Genetics, Cell Biology and Development, University of Minnesota, Minneapolis, Minnesota 55455,; the § Biology Department and Rosenstiel Basic Medical Sciences Research Center, Brandeis University, Waltham, Massachusetts 02454, and; the ¶ Institute of Molecular and Cell Physiology, Medical School, Hannover, 30625 Hannover, Germany

**Keywords:** Calcium-binding Protein, Cell Division, Chemotaxis, Dynein, Epilepsy, Intermediate Filament, Tubulin

## Abstract

Cilia and flagella are conserved, motile, and sensory cell organelles involved in signal transduction and human disease. Their scaffold consists of a 9-fold array of remarkably stable doublet microtubules (DMTs), along which motor proteins transmit force for ciliary motility and intraflagellar transport. DMTs possess Ribbons of three to four hyper-stable protofilaments whose location, organization, and specialized functions have been elusive. We performed a comprehensive analysis of the distribution and structural arrangements of Ribbon proteins from sea urchin sperm flagella, using quantitative immunobiochemistry, proteomics, immuno-cryo-electron microscopy, and tomography. Isolated Ribbons contain acetylated α-tubulin, β-tubulin, conserved protein Rib45, >95% of the axonemal tektins, and >95% of the calcium-binding proteins, Rib74 and Rib85.5, whose human homologues are related to the cause of juvenile myoclonic epilepsy. DMTs contain only one type of Ribbon, corresponding to protofilaments A11-12-13-1 of the A-tubule. Rib74 and Rib85.5 are associated with the Ribbon in the lumen of the A-tubule. Ribbons contain a single ∼5-nm wide filament, composed of equimolar tektins A, B, and C, which interact with the nexin-dynein regulatory complex. A summary of findings is presented, and the functions of Ribbon proteins are discussed in terms of the assembly and stability of DMTs, ciliary motility, and other microtubule systems.

## Teaser

**Background:** Ciliary microtubules contain hyperstable Ribbons of adjoining protofilaments.

**Results:** Using echinoderm flagella, the locations of Ribbons, tektins, and Ca^2+^-binding proteins (related to human epilepsy) are studied biochemically and by immuno-cryo-electron tomography.

**Conclusion:** The locations of these proteins create a biochemically, structurally unique region of ciliary A-microtubules.

**Significance:** The results indicate specialized functions for Ribbons, with potential roles in assembly, motility, and/or signal transduction.

## Introduction

Cilia and flagella (cilia will also refer generally to flagella) evolved very early in eukaryotic history (1) and are conserved in their polypeptide composition, structure, and function as motile and sensory organelles in eukaryotes, including humans, wherein these organelles are directly involved in multiple diseases and developmental disorders (2). Much is known about the mechanisms of signaling and trafficking between cytoplasm and cilium (3, 4) and about the structure-function relationship of basal bodies (5–7) and motile ciliary axonemes (8, 9). The least well understood part of the ciliary machinery is the 9-fold array of axonemal doublet microtubules (DMTs)^4^ that are remarkably stable, compared with cytoplasmic microtubules (MTs), and that serve as dynamic scaffolds for the attachment of hundreds of effector proteins (10, 11).

We wish to understand the basis of the extreme stability of DMTs, their highly complex structure, and how these properties function in ciliary assembly, motility, and signaling. The starting point comes from the early observations that ciliary MTs from sources as diverse as protists (*Chlamydomonas*), sea urchin sperm, and mammalian respiratory epithelia contain hyper-stable “Ribbons” of three to four adjoining protofilaments (12–16). Ribbons contain two important classes of nontubulin proteins that we study in this report (Table 1) (9, 17–45).

**TABLE 1:**
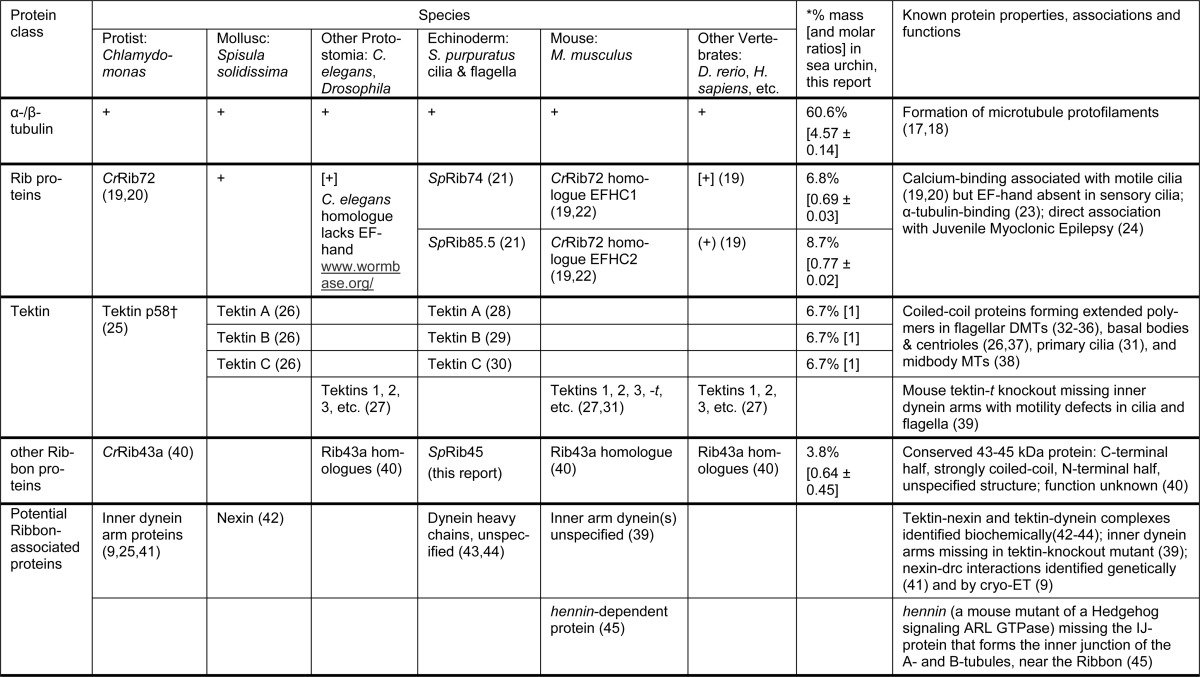
**Proteins associated with or forming stable protofilaments of doublet microtubules in various species** Each horizontal row corresponds to one class of proteins. + means protein is present; (+) means present in genome, gene product was predicted. For the sea urchin, % mass (and molar ratios) is based on densitometry of SDS-PAGE Serva Blue integrated staining intensity (÷ molecular mass) (Fig. 5*b*) normalized to tektin C = 1.0; standard deviations reflect the error in three measurements; the exact % and ratios will depend on the unknown dye-binding ratios (intensity/mg) of unrelated proteins.

† Tektins are contained within Sarkosyl-stable Ribbons from sea urchins and mammals but not from *Chlamydomonas.* For the nomenclatures and sequence relationships, see Ref. 27.

The first class consists of calcium-binding (Rib) proteins, which are related to the cause of juvenile myoclonic epilepsy. The first identified representatives of this class, *Sp*Rib74 and *Sp*Rib85.5 (formerly Sp77 and Sp83, respectively), were characterized as proteins bound to Ribbons of sea urchin *Strongylocentrotus purpuratus* sperm flagella and to echinoderm and mammalian basal bodies and centrioles (21, 46, 47). The first-to-be-sequenced Rib protein of *Chlamydomonas reinhardtii* flagella, *Cr*Rib72, contains three DM10 domains and two EF-hand calcium-binding domains (19, 20). Rib protein homologues have since been identified in cilia, basal bodies, and centrosomes of other organisms (48, 49) (wormbase.org; zfin.org). Subsequently, human *RIB* homologue genes, *EFHC1* and *EFHC2*, were shown to cause juvenile myoclonic epilepsy when mutated (24, 50). We refer to members of this class of Ribbon proteins as follows: group 1, *Cr*Rib72, *Sp*Rib74, mouse *Mm*EFHC1, human *Hs*EFHC1, etc.; and group 2: *Sp*Rib85.5, *Mm*EFHC2, etc.

The second class of Ribbon-associated proteins consists of tektins. These ∼50-kDa proteins have been characterized extensively from echinoderm and molluscan cilia (30, 32, 33, 35, 36, 51), and they have been identified in genomes ranging from protists to humans (27, 30). Tektins are expressed in mouse olfactory epithelial cells and photoreceptors that contain nonmotile, primary cilia (31), but they have not been found by proteomic studies of primary cilia of mouse kidney cells (52). Tektins have been localized to centrosomes, centrioles, basal bodies, and mitotic spindles in species from *Chlamydomonas* to human (26, 37, 48, 53–56). Interestingly, in mammalian cells tektins and the Rib homologue *Mm*EFHC1 are both associated with cytokinesis midbody MTs (38, 56, 57), and *Mm*EFHC1 affects neuronal development (24). Thus, it is unclear whether the etiology of juvenile myoclonic epilepsy rests with motile or nonmotile ciliopathies, with cell division defects, and/or with other nonciliary roles of *RIB* genes. Finally, biochemical, genetic, and structural evidence indicates an interaction between tektin and dynein (see under “Discussion”).

Three tektins (A, 53 kDa; B, 51 kDa; and C, 47 kDa) are major components of Ribbons from sea urchin axonemal MTs and can be isolated as Sarkosyl-urea insoluble filaments 5 nm wide and fibrils 2 nm wide (15, 32, 33). They form coiled-coil subunits, predicted to be between 32 and 48 nm long, with tektins A and B forming heterodimeric filaments and tektin C forming homo-oligomers (30, 34, 44). Tektins extend the length of DMTs (32, 34, 37), but it has been unclear whether tektin C associates with tektin AB filaments or forms filaments that are spatially separated from AB filaments (27).

**FIGURE 1.**
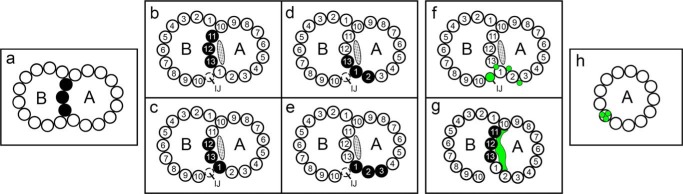
**Summary of previously proposed locations of Ribbons of PFs.** PFs (*black*) and tektin fibrils/filament (*green*) are shown. *A*, A-tubule; *B*, B-tubule; *IJ*, inner junction; PFs numbered according to Ref. 58. The approximate location and shape of the partition-associated material (*stippled*) is redrawn from Ref. 59 and likely contains the MIP4 density (11). *a*, the 3-PF Ribbon was originally suggested to arise from the PFs forming the partition between the A- and B-tubules (12). *b–e*, given the unknown structure of the A-tubule/B-tubule junctions prior to tannic acid fixation (60) and the lack of three-dimensional information about the origin of these PFs, it was uncertain whether the stable Ribbon corresponded to PFs A10-11-12 (data not shown) or to PFs A11-12-13 (*b*), or PFs A12-13-1 (*c*), or whether there were two kinds of Ribbons. Several observations pointed to a different or second location of Ribbon PFs closer to the inner dynein arms (see “Discussion”), *e.g.* PFs A13-1-2 (*d*) or PFs A1–3 (*e*). *f–h*, different locations have also been proposed for tektins, *i.e. f*, the IJ structure or thin fibrils either between PFs or in the grooves between PFs (15, 27, 32, 61), or *g*, the partition material on the A-lumen side of tubulin PFs A11-12-13-1 (redrawn from Ref. 62), or *h*, one of the PFs of the Ribbon (15, 44, 63).

The functions of Ribbon proteins will depend on their interactions in DMTs; however, the number and types of Ribbons, their composition, and the location of Ribbons and tektins within DMTs have been controversial, as described in Fig. 1 (11, 12, 15, 27, 32, 44, 58–63). Here, using biochemical fractionation techniques, proteomics, immunoelectron microscopy, cryo-electron tomography (cryo-EM), and a new, integrative approach of immuno-cryo-EM/ET, we have localized *Sp*Rib74 and *Sp*Rib85.5, resolved the location of the Ribbon of four stable protofilaments in flagellar DMTs, and furthered our understanding of tektin filament structure and location. Cryo-ET combined with subtomogram averaging is a state-of-the-art imaging technique and allows for the determination of three-dimensional structural arrangements at about 3 nm resolution (8, 64, 65).

## EXPERIMENTAL PROCEDURES

### 

#### 

##### Preparation of Biological Specimens

Sea urchins (from Marinus Scientific) were spawned into artificial sea water (Marine Biological Laboratory, Woods Hole, MA), and sperm cells were filtered through cheesecloth.

Flagellar axonemes were purified (59) by adding 1 mm PMSF to the Triton homogenizing solution. DMTs were purified (59) by dialysis against 10TEAD (10 mm Tris, pH 7.8, 0.1 mm EDTA, 0.01% NaN_3_, 1 mm DTT).

A-tubules for quantitative biochemical analysis were prepared by thermally fractionating DMTs (40 °C for 5 min (66)). DMT→Ribbon transitions for cryo-EM/ET analysis were prepared by extending the heating to 10 min and for negative staining by partial Sarkosyl extraction.

Sarkosyl Ribbons were purified (67), using 0.5% Sarkosyl (Hamposyl L-95; W. R. Grace, Nashua, NH), and dialyzed into 10TEAD. Tektin filaments were purified (67), using 0.5% Sarkosyl and 1–3 m urea in 10TEAD.

Ribbon→filament transitions were prepared by mixing Ribbons with an equal volume of Sarkosyl-urea (above) and then diluting with 10TEAD. The sample was collected by centrifugation (100,000 × *g* for 20 min), resuspended, and dialyzed against 10TEAD.

##### Biochemical Procedures

Protein concentrations were measured by the Micro BCA procedure (ThermoScientific).

SDS-PAGE was conducted as described (59). Gels were either electroblotted for immunostaining (below) or stained for quantitation in 0.0175% Serva Blue R, 25% 2-propanol, and 10% acetic acid overnight followed by 0.00175% Serva Blue, 10% 2-propanol, and 5% acetic acid for 5 h, destained with four changes of 5% acetic acid over 2 days, and immediately scanned for densitometry (see below).

Immunoblotting (68) was conducted using the following: Immobilon-P (Millipore) or nitrocellulose (Bio-Rad); CHAPS and SuperBlock (ThermoScientific); alkaline phosphatase-conjugated goat anti-rabbit or alkaline phosphatase goat anti-mouse antibody (Promega); and nitro blue tetrazolium and 5-bromo-4-chloro-3-indolyl phosphate (Sigma).

##### Quantitative Gel Densitometry

Defined amounts of *S. purpuratus* B(αβ)-tubulin, purified to >99% (59), were electrophoresed; gels were stained and destained as above. Gels were digitized as tiff files with an Epson V750 Pro scanner. A transparent step wedge (0.20 density steps; part no. T2120CC; Stouffer Graphics Arts, Mishawaka, IN) and ImageJ (rsbweb.nih.gov) were used to integrate the stain densities of selected polypeptides. The ratio of tubulin/stain intensity was quantifiable from 0.2 to 10 μg/subunit/SDS-PAGE lane. Molar ratios were calculated by dividing the integrated staining intensity of a given polypeptide band by its molecular mass.

Two-dimensional IEF/SDS-PAGE was performed (69), using 8 m urea, 2% CHAPS, 0.4% DTT, and 0.5% IPG buffer, and separated on 13-cm immobilized pH 3–10 nonlinear gradient dry strips (GE Healthcare) for 34–44 kV-h, followed by 10% SDS-PAGE, and stained with Coomassie Brilliant Blue G-250 (Sigma). Mass spectrometry analysis was performed as described previously (70).

##### Antibody Preparation, Characterization, and Purification

Nitrocellulose strips of *Sp*Rib74 and *Sp*Rib85.5 were sent to Pocono Rabbit Farm and Laboratory and used to immunize rabbits according to company procedures. Preimmune sera and antisera were tested by immunoblotting against DMTs. Antibodies were affinity-purified as described previously (71).

Other antibodies used are as follows: individually specific rabbit antibodies against *Lytechinus pictus* tektins-A, -B, and -C, affinity-purified (37); rabbit antiserum against the tektin consensus sequence RPNVELCRD (44); and mouse monoclonal antibody specific for acetylated α-tubulin from wide ranging species (53, 54, 72). All antisera and purified antibodies were characterized for their titers and specificities by analytical immunoblotting as described above.

##### Electron Microscopy

Negative staining for EM (73) was performed using 1% aqueous uranyl acetate. Immuno/negative staining was conducted (32) using 6-nm gold-conjugated goat anti-rabbit (F_ab_), 10-nm gold-conjugated goat anti-rabbit (F_ab_), and 10-nm gold-conjugated goat anti-mouse (F_ab_) (Electron Microscopy Sciences).

##### Measurements of Immunogold Labeling

Gold particles were counted only if they were within 25 nm of Ribbons or filaments; the nonspecific background was very low. From these counts, the percentage of particles bound to Ribbons or filaments was calculated. Particles that were ambiguously associated with both Ribbons and filaments, or with debris, were not counted.

For immuno-cryo-EM and immuno-cryo-ET, the following procedure was adopted. Samples were prepared as for immuno/negative staining and applied to EM grids coated with continuous carbon films, except that the last wash with 10TEAD and the negative staining were omitted; instead, the final drop of gold-labeled secondary antibody was wicked with blotting paper, leaving residual gold particles as fiducial markers (for the tilt series alignment process); immediately 4 μl of 10TEAD was added to the grid, which was then cryo-immobilized for cryo-EM/ET as described below.

For cryo-EM and cryo-ET imaging, Quantifoil grids (Quantifoil MicroTools GmbH) with holey carbon support film (copper; 200 mesh; R2/2) were used. Prior to use, they were glow-discharged for 30 s at −40 mA. 3 μl of sample and, as needed, 1 μl of 10× concentrated 10-nm colloidal gold solution (Sigma) were applied and slightly mixed on the grid before excess liquid was blotted with filter paper. Immediately, the grid was plunge-frozen into liquid ethane and stored in liquid N_2_ for later use.

For cryo-ET and image processing (74), cryo samples were imaged at 300 kV on an F30 electron microscope (Tecnai F30; FEI, Inc.) equipped with a postcolumn energy filter (Gatan, Inc.) operated in zero loss mode with a 20-eV slit width. Images were collected on a 2k × 2k CCD camera (Gatan, Inc.), with single-axis tilt series recorded within a tilt range from −66 to 66° with 1.5–2.5° increments, a defocus of −6 to −8 μm, and a total accumulated dose <100 e/Å^2^ by using the microscope software SerialEM (75). Nominal magnification was either 13.5k or 22.5k, resulting in a pixel size of 1 or 0.556 nm. The tilt series were aligned and reconstructed into tomograms using the IMOD package (76). Structural repeat units were chosen, aligned, and averaged using the software PEET (8, 9). Resolutions of the structures were measured using the 0.5 criterion of the Fourier Shell Correlation method. The UCSF Chimera package (77) was used for three-dimensional visualization and isosurface rendering.

## RESULTS

### 

#### 

##### Structure of DMTs

**FIGURE 2.**
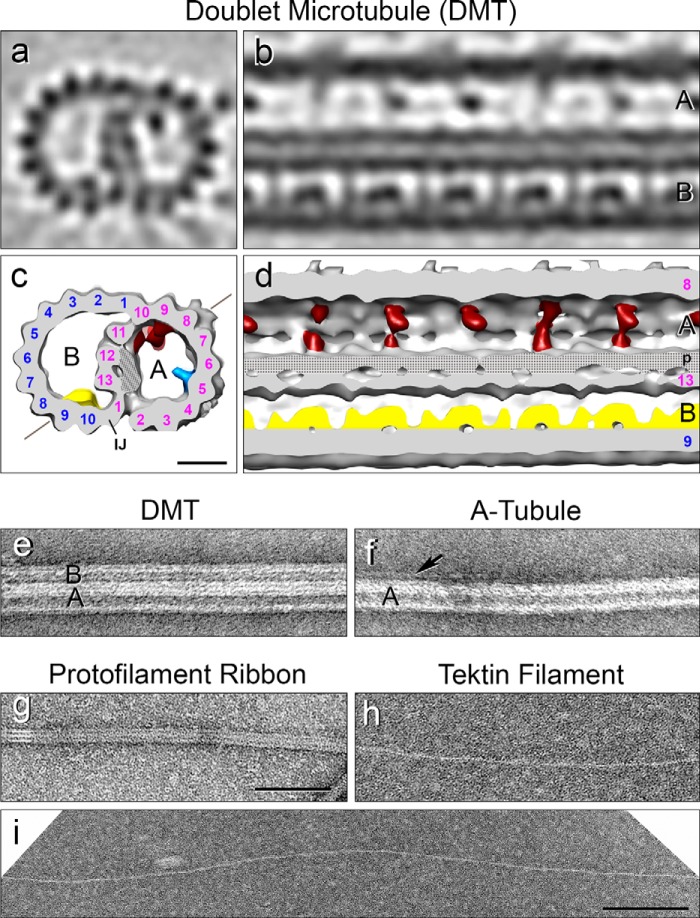
**Structure of intact and fractionated DMTs.** *a–d*, three-dimensional structure of an intact sea urchin (*S. purpuratus*) flagellar DMT obtained by cryo-ET and subtomogram averaging of the 96 nm axonemal axial repeats (1200 repeats; resolution in the center of the volume is 3.3 nm). The DMT is shown as tomographic slices (*a* and *b*) and isosurface renderings (*c* and *d*) in cross-sectional (*a* and *c*) and longitudinal (*b* and *d*) views. For clarity, the dynein arms and radial spokes were removed, and only the DMT core is shown. *A*, A-tubule; *B*, B-tubule; PFs are numbered; *IJ*, inner junction protein(s); microtubule inner protein MIP1, *blue*; MIP2, *red*; MIP3, *yellow*; *stippled area* marks the thickened, non-tubulin partition-associated material (*p*); diagonal line in *c* demarcates the viewing angle in *b* and *d*. DMTs and fractions are either viewed from the proximal (−) to the distal (+) end of the DMT in cross-section (*a* and *c*) or oriented with the proximal end to the left (*b* and *d*). *Scale bar*, 10 nm for *a–d*. See also supplemental Movie S1. *e–i*, negative stain EM specimens (*S. purpuratus*) corresponding to the fractions shown in Fig. 3: *e*, DMT composed of A- and B-tubules. *f*, A-tubule with remnant PF(s) (*arrow*) of the B-tubule corresponding to PFs B9 and B10 that are tightly associated with the A-tubule by the IJ proteins. *g*, Sarkosyl Ribbon typically composed of three PFs here (*white lines*) or four PFs from *L. pictus* (data not shown). *h* and *i*, tektin filaments measuring ∼5 nm wide and up to several microns long, composed of tektins A, B, and C (Fig. 4*a*, *lane 3*). Note that tektin filaments appear to be smooth, with no apparent lateral side projections. *Scale bars*, 100 nm for *e–h*, 200 nm for *i*.

Cryo-ET of intact sea urchin sperm flagella provided the highest resolution (3.3 nm) three-dimensional structure to date of sea urchin flagellar DMTs by subtomographic averaging (Fig. 2, *a–d*), where small volumes containing repeating macromolecular complexes are extracted from the three-dimensional tomographic reconstruction, aligned with each other in three-dimensional space, and then averaged. This structure was later used for correlation with the three-dimensional structures of fractionated DMTs. We found that, in particular, the electron density associated with the lumenal surface of the partition PFs, A11-12-13-1, is more complex than previously thought and consists of several distinct masses that connect periodically to the protofilaments (supplemental Movie S1).

##### Quantitative Fractionation of the Axoneme and Distribution of PF-Ribbon Proteins

**FIGURE 3.**
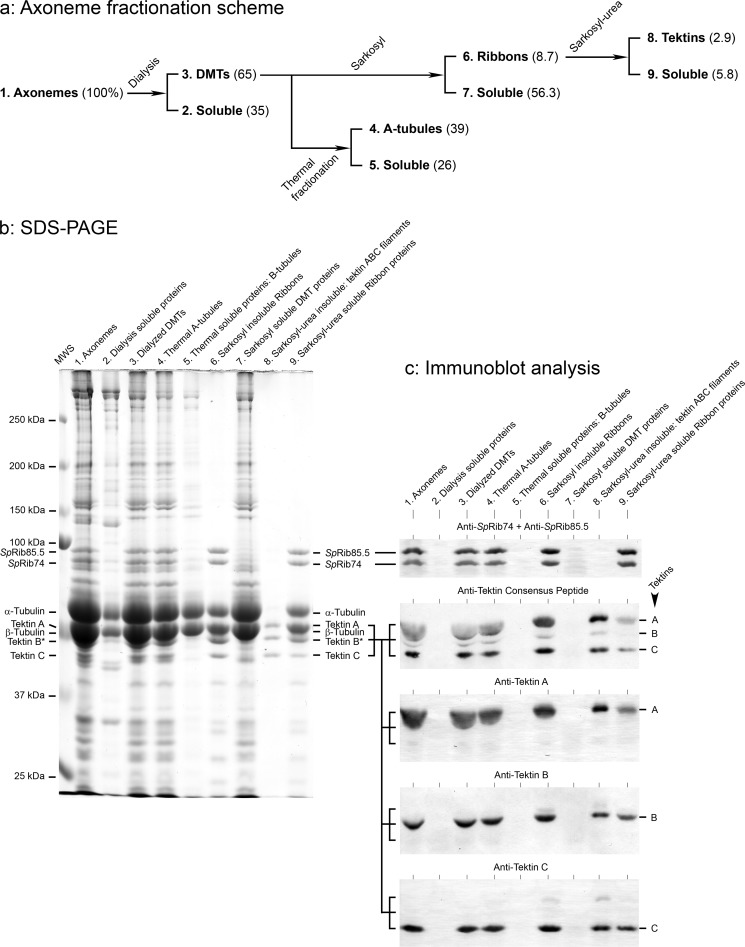
**Quantitative fractionation of *S. purpuratus* axonemes and distribution of Ribbon proteins.** *a*, axonemes were sequentially fractionated in discrete steps, and matched fractions were analyzed by SDS-PAGE (*b*), immunoblotting (*c*), and EM (Fig. 2). *Numbers* in parentheses are the percentages of protein in each fraction relative to axonemes (100%), referenced to BSA. *b*, SDS-PAGE of the fractions in *a*. The *asterisk* (Tektin B*) indicates the presence of co-migrating *Sp*Rib45, a homologue of *Cr*Rib43a (Fig. 4*b*). *MWS*, molecular weight standards. *c*, immunoblot analysis of identical replicas of the gel lanes in *b*, stained with the following antibodies (characterized in Fig. 4*a*): anti-*Sp*Rib74 + anti-*Sp*Rib85.5 (a mixture of the two separately specific antibodies); anti-tektin consensus peptide against the sequence RPNVELCRD, present in most tektins from echinoderms to humans; anti-tektin A; anti-tektin B; and anti-tektin C. The anti-consensus peptide shows that no polypeptides with this peptide, other than tektins A, B, and C, are present in any of the fractions; the uneven staining of the different tektins may be due to different and possibly interfering amino acid residues bordering the consensus sequence in the full-length polypeptide chains (30). Results: by densitometry, >95% of *Sp*Rib74/85.5 are retained in the Ribbon fraction (*lane 6*) but are completely solubilized along with all tubulin upon Sarkosyl-urea extraction (*lane 9*); and >95% of tektins are retained in Ribbons (*lane 6*). In the end, when Ribbons are extracted with Sarkosyl-urea, the resulting insoluble filaments (*lane 8*, Fig. 2*h*) are composed of tektins A, B, and C in equal molar amounts (Fig. 5*a*); a fraction of these tektins become soluble (*lane 9*). Tektin A and B bands are distorted and do not line up precisely in the heavily loaded lanes, because they are “pushed” ahead by the larger amount of nearly co-migrating tubulin.

As described in Fig. 1, the potential number of Ribbons in a DMT is unknown, *i.e.* there could be one or more than one Ribbon per DMT. Equally important, the amount of the proteins associated with Ribbons has never been accurately documented, and thus it has been uncertain whether the tektins and the Ca^2+^-binding proteins are specifically restricted, *e.g.* each to a different type of Ribbon, or whether tektins and/or the Ca^2+^-binding proteins are also present in other domains of ciliary MTs. To this end, we quantitatively fractionated axonemes into discrete entities, *i.e.* DMTs, A-tubules, Ribbons, and tektin filaments that were evaluated by EM to be >95% homogeneous (Fig. 2, *e–i*), and we analyzed these fractions biochemically. The fractionation scheme in Fig. 3*a* provides a map for interpreting the results to follow.

##### Stoichiometry of Tektins in Isolated Filaments

**FIGURE 4.**
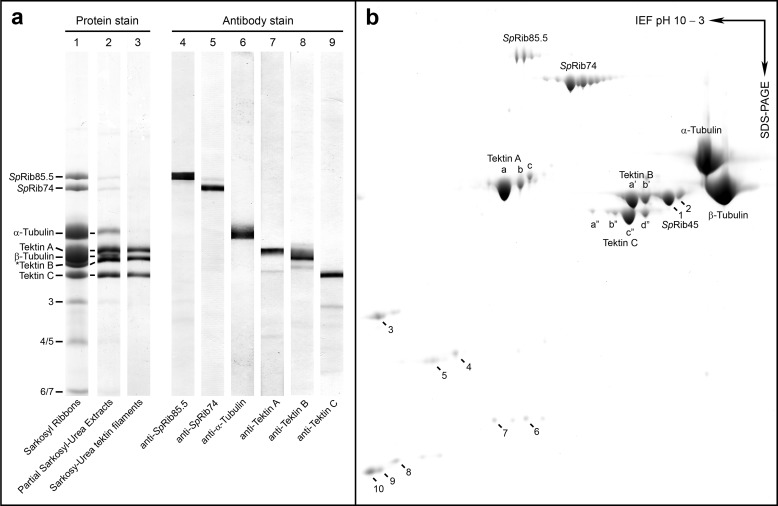
**Composition of Ribbons, characterization of antibodies, and two-dimensional PAGE for MS analysis.** *a, lanes 1–3*. SDS-PAGE protein staining of the following DMT fractions: *lane 1, S. purpuratus* Sarkosyl Ribbons (EM appearance shown in Fig. 2*g*) showing the principal constituent polypeptides; *lane 2*, ribbons partially extracted with urea to reduce tubulin that perturbs and obscures nearby bands; *lane 3*, Sarkosyl-urea-purified tektin filaments (Fig. 2*h*) composed of tektins A, B, and C. Masses as determined from their sequences are as follows: *Sp*Rib85.5, 85.5 kDa; *Sp*Rib74, 74 kDa; tektin A, 53 kDa; tektin B, 51 kDa; tektin C, 47 kDa; α- and β-tubulin, ∼50 kDa (*SpRib45 co-migrates with tektin B). *Lanes 4–9* show immunoblots of *S. purpuratus* DMTs stained with the indicated antibodies. *b*, two-dimensional IEF/SDS-PAGE of Sarkosyl Ribbons. Major polypeptides (labeled) and spots 1–10 were reproducibly present and were cut out and analyzed by MALDI-TOF mass spectrometry (Table 2). Spots 1 and 2 were identified as *Sp*Rib45, homologue of *Chlamydomonas Cr*Rib43a. Corresponding positions of spots 3, 4/5, and 6/7 are indicated in *a*.

**FIGURE 5.**
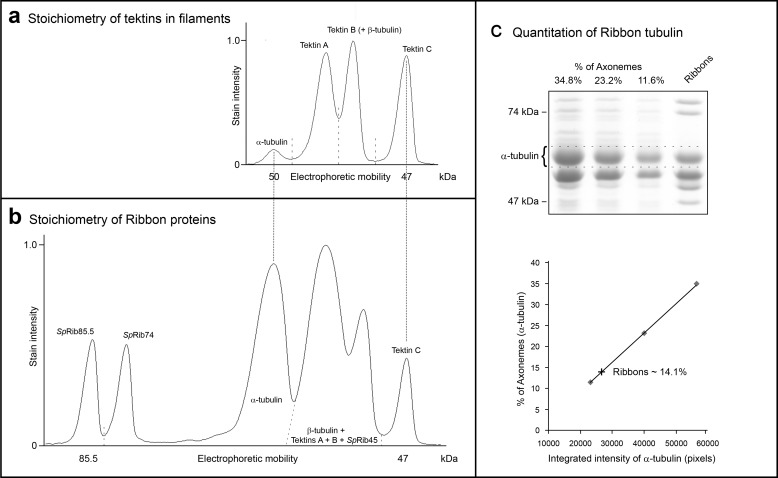
**Stoichiometry of Ribbon proteins.** *a*, stoichiometry of tektins. Ribbons were extracted once with Sarkosyl-urea, leaving a residual amount of associated tubulin but minimizing the loss of tektin C, resolved by SDS-PAGE, and stained quantitatively with Serva Blue. Gel lanes were scanned, and the stain intensities of α-tubulin and tektins A, B, and C measured. Because β-tubulin migrates closely with tektin B (Fig. 4*a*, *lane 2*) and because the moles of α-tubulin must equal the moles of β-tubulin, the stain intensity of α-tubulin was subtracted from the intensity of the (tektin B + β-tubulin) peak to determine the amount of tektin B alone. The intensities of tektins A, B, and C were divided by their masses (53, 51, and 47 kDa, respectively) to give intensity/kDa and normalized to tektin A. The molar ratio of tektins A/B/C was thus determined to be 1:1:1. *Dashed lines* indicate the separation of the intensities of the individual polypeptides, and the *dotted lines* indicate the registration of the respective polypeptides in *b. b*, stoichiometry of Ribbon proteins. DMTs were extracted once with Sarkosyl (to minimize sample loss) and analyzed as described for tektin filaments above. *Dashed lines* indicate the separation of the intensities of the individual polypeptides. The stoichiometry of the Ribbon proteins was thus calculated and reported in Table 1. *c*, the number of Ribbons per axoneme was estimated as follows. Reference lanes were loaded with the amount of axonemal tubulin calculated for one, two, and three Ribbons (of three tubulin PFs) per DMT (*i.e.* 11.6, 23.2, and 34.8%, respectively), against which the experimental Ribbon sample was compared. The α-tubulin region was measured (*bracket, dashed lines*) and plotted as % of axonemal α-tubulin *versus* the integrated (stain) intensity of α-tubulin, ■. The experimentally obtained Ribbon α-tubulin (+) corresponds to ∼14% of the axonemal α-tubulin, very close to the amount (11.6%) expected for one Ribbon of three tubulin PFs per DMT. The value 14.1% is probably artificially high and closer to the theoretical 11.6%, because (i) central pair-MTs are less stable than DMTs and therefore some central pair-tubulin is lost during the isolation of axonemes, and (ii) because a small percentage (<5%) of the once-extracted Ribbons contains four PFs and not three.

Initially, to more accurately define tektin filaments, we analyzed their composition and structure (Fig. 2*h*; Fig. 4*a, lane 3*; Fig. 5). We know from previous studies that tektins A and B exist in the filament polymer as AB heterodimers (34) and that tektin C, being slightly more soluble than tektins A and B (67), is lost in some filament preparations. Thus, earlier quantitation studies (33) have been questioned (27). Here, by lowering the concentration of urea to a level (1.75 m) sufficient to solubilize tubulin but retain tektin C, we obtain filaments in which the molar stoichiometry of A:B:C is 1:1:1 (Fig. 5*a*). Furthermore, tektin filaments so obtained appear to be intact, with a constant width, with no evidence of untwisting over distances of several microns, and smooth with no lateral side projections (Fig. 2*i*).

##### Quantitation of Ribbon Proteins

Next, we measured the molar ratio of Ribbon proteins. Isolated Ribbons from *S. purpuratus* contain polypeptides *Sp*Rib85.5, *Sp*Rib74, tektins A, B, and C (Fig. 3*c*, *lane 6*), along with acetylated α-tubulin, β-tubulin, and *Sp*Rib45, a newly identified homologue of *Chlamydomonas* Rib43a (Fig. 4, *a* and *b*; Table 2) (40). Ribbons also contain at least four smaller polypeptides (20–40 kDa) of low abundance (perhaps similar to those reported in *Chlamydomonas* (78)) that we identified by mass spectrometry but did not further study (Fig. 4*b*; Table 2). However, it should be noted that the composition of sea urchin Ribbons does differ in important ways from that of *Chlamydomonas* Ribbons (see under “Discussion”) (20, 25, 40). The stoichiometry of Ribbon proteins has been difficult to determine due to the similar masses and hydrophobicities of tektins and tubulins (33). Although these proteins separate well by two-dimensional IEF/SDS-PAGE (Fig. 4*b*), their stoichiometry was not reproducible (perhaps due to the ampholytes and/or to errors in summing the widely spread spots). Instead, we calculated their stoichiometry by quantitative SDS-PAGE densitometry (Fig. 5*b* and Table 1). To summarize, *Sp*Rib74 and *Sp*Rib85.5 comprise ∼16% of the Ribbon protein, tektins ∼20%, and tubulin ∼60%; their approximate molar ratio (in parentheses) is as follows: α-tubulin (4.6), β-tubulin (4.6), *Sp*Rib74 (0.7), *Sp*Rib85.5 (0.8), *Sp*Rib45 (0.6), tektin A (1), tektin B (1), and tektin C (1).

**TABLE 2:** **Identification of Ribbon proteins in *S. purpuratus* flagellar axonemes by MALDI-TOF MS** Spots that were identified as the same protein are grouped. A Mascot score of >70 is considered significant (*p* < 0.05). Scores were obtained from a search with MASCOT Peptide Mass Fingerprint at: http://www.matrixscience.com/cgi/search_form.pl?FORMVER=2&SEARCH=PMF

Accession no.	Protein	Theoretical mass	Mascot score	Sequence coverage	Spot no.
		*Da/pI*		%	
gi|72153570	RIB43A-like with coiled-coils protein 2-like	45,226/5.36	207	51	1
			153	46	2
gi|72083424	Hypothetical protein	37,122/8.87	112	35	3
gi|390357925	Uncharacterized protein LOC577943	34,940/8.14	178	44	4
			160	43	5
gi|390343367	Uncharacterized protein C9orf135-like	28,162/7.16	76	26	6
			108	33	7
gi|72130598	UPF0573 protein C2orf70 homolog A-like isoform 2	23,789/9.05	90	39	8
			128	59	9
			165	64	10

##### Distribution of Ribbon Proteins

Before localizing the Ribbon proteins by EM, we quantitated their presence or absence in the various axoneme fractions by immunoblotting (Fig. 3*c*). The specificities of the antibodies were critical for EM localization and were therefore thoroughly tested (Fig. 4*a*). Each antibody showed nearly complete specificity for its respective antigen in DMTs (with only slightly detectable cross-reaction with related proteins or with small levels of proteolytic fragments of their respective antigens). Important to our immuno-EM of tektin C below, anti-tektin C shows no detectable cross-reaction with tektins A or B (Fig. 4*a, lane 9*). These specificities were similar whether the antibodies were affinity-purified or not, but monoclonal or affinity-purified antibodies were generally used throughout this study.

For quantitative analysis, all nine of the fractions shown in Fig. 3*b* were blotted onto five Immobilon sheets, and each sheet was stained with a different antibody (Fig. 3*c*). The major results of this analysis are as follows: (i) >95% of *Sp*Rib74 and *Sp*Rib85.5 (by densitometry) is retained in the Sarkosyl Ribbon fraction derived from DMTs or A-tubules, and these proteins are completely solubilized by the subsequent Sarkosyl-urea extraction; these antigens are not or barely detectable in the other soluble fractions; and (ii) tektins A, B, and C are present exclusively in the Ribbons derived from DMTs or A-tubules and are largely retained in the Sarkosyl-urea insoluble filament fraction, with a portion of them being solubilized by Sarkosyl-urea.

##### Localization of the Stable PF-Ribbon and Associated SpRib74/SpRib85.5

Because of the uncertainty and controversies over the location(s) of Ribbons within the DMT (Fig. 1), we considered the possibility that there might be two classes of Ribbons as follows: an *Sp*Rib74/*Sp*Rib85.5-containing Ribbon and a tektin-containing Ribbon. Attempts were made to separate two such potential classes of Ribbons by immunoprecipitation and by sucrose density sedimentation, but neither method separated populations of chemically distinct Ribbons. Instead, we quantitated the amount of axonemal tubulin recovered in the Ribbon fraction (Fig. 5*c*), and we found that there is only sufficient tubulin present in the Ribbon fraction to account for one Sarkosyl-stable Ribbon per DMT.

We also considered the possibility that Ribbons might derive from central pair MTs. Indeed, central pair MTs disassemble initially into Ribbons during the dialysis purification of DMTs but then dissolve completely (13, 59), because Ribbons are not observed by EM in dialysis-purified DMTs before Sarkosyl extraction and because the dialysis supernatant contained no detectable Ribbon proteins (Fig. 3*c*, *lane 2*). Thus, our results demonstrate that Sarkosyl-stable Ribbons derive almost entirely, if not exclusively, from DMTs, and the greater lability of central pair MT Ribbons must be attributed to other factors.

**FIGURE 6.**
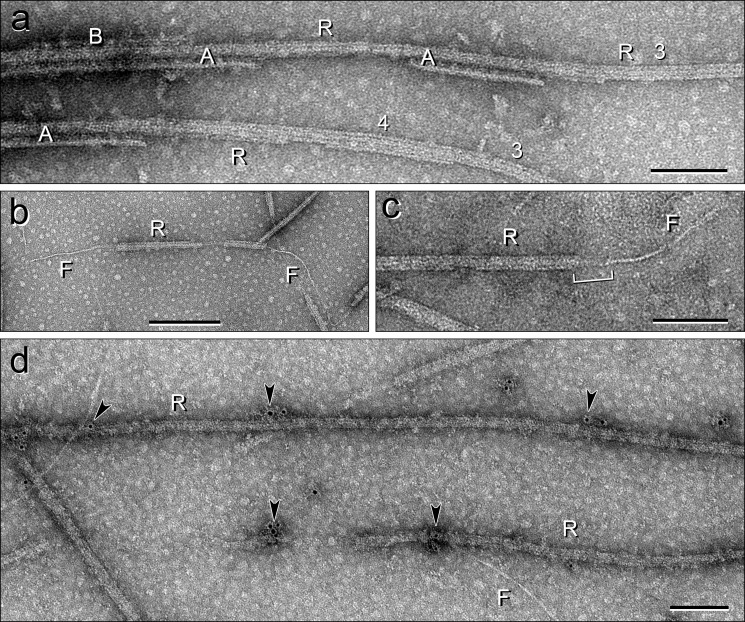
**Negative stain-EM and immuno-EM of DMT, Ribbon, and filament fractions.** *a*, negatively stained preparation of a partially extracted DMT, showing a transition from DMT→A-tubule→Ribbon→A-tubule→Ribbon and one transition from A-tubule→Ribbon. *A*, A-tubule; *B*, B-tubule; *R*, Ribbon of four and then three PFs. *b* and *c*, Ribbon→filament transitions, negatively stained, showing the emergence of a single ∼5-nm wide tektin filament. *R*, Ribbon; *F*, filament. *c, bracket* shows the region of the transition, where the origin of the filament in the Ribbon is obscured (see also cryo-ET in Fig. 12); this could be due to remaining *Sp*Rib74, *Sp*Rib85.5, and/or tubulin adhering to the stable tektin filament as it emerges from the Ribbon. *d*, purified Ribbons (*R*) labeled with anti-*Sp*Rib85.5 primary antibody/gold-secondary antibody and negatively stained for EM. Some of the gold particles are indicated by *arrowheads*. Note that the tektin filaments (*F*) appearing in the field are not labeled with gold antibody. *Scale bars, a, c*, and *d*, 100 nm; *b*, 200 nm.

**FIGURE 7.**
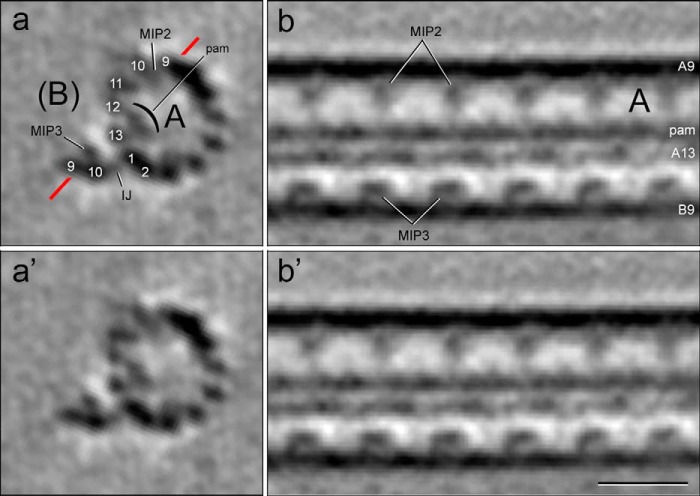
**Cryo-electron tomogram of a thermally fractionated, reconstructed, and averaged DMT.** Labeled and unlabeled cross-slices (*a* and *a′*) and longitudinal slices (*b* and *b′*) of an A-tubule with B-tubule hook are shown. Plane of *b* and *b′* is indicated by *red line* in *a. a* and *a′*, viewed from the proximal (minus) end to the distal (plus) end; *b* and *b′*, proximal end to the *left.* Shown are the following: A-tubule (*A*, with PFs A9 to A2 numbered); *MIP2*; *pam/bracket*, partition-associated material; and the remaining portion of the B-tubule (*B*), including the inner junction component(s) (*IJ*), PFs B9–10 and MIP3. These markers were used to identify the location of the stable protofilaments A11-12-13-1 (see Fig. 8). *Scale bars, a, a′, b*, and *b′*, 20 nm.

**FIGURE 8.**
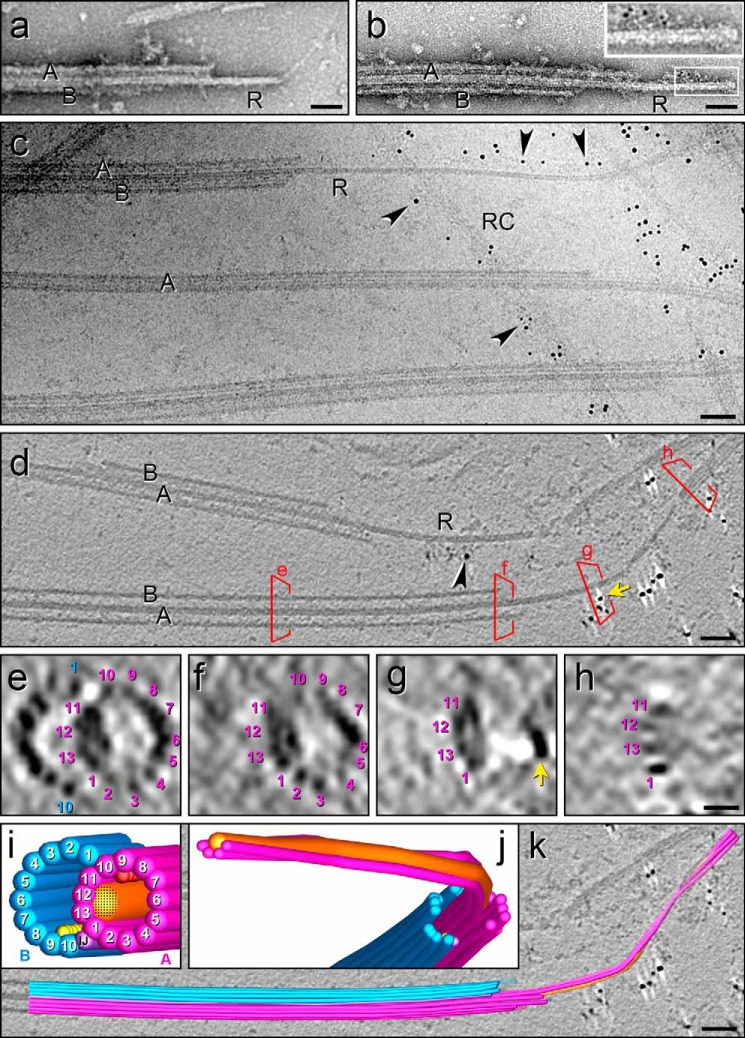
**Localization of the stable ribbon and *Sp*Rib74.** *a* and *b*, negatively stained DMT→Ribbon transitions without (*a*) and with anti-*Sp*Rib74/immunogold labeling (*b*); *inset* at higher magnification. Anti-*Sp*Rib74 does not label intact DMTs or A-tubules, and instead it labels only the extending Ribbon along the side of the Ribbon facing the lumen of the A-tubule. *c–h*, immuno-cryo-EM (*c*) and immuno-cryo-ET (*d–h*) of DMT→Ribbon transitions after labeling with anti-*Sp*Rib74/immunogold (*arrowheads*). Isolated Ribbons (*RC*) that were added as a control are continuously labeled by the antibodies (see also Fig. 9). In DMT→Ribbon transitions, labeling only occurs along Ribbons and only along the side of the Ribbon facing the lumen of the A-tubule. *d*, *red boxes* and *letters e–h* along the DMT→Ribbon transition indicate where the cross-sectional slices in *e–h* are taken. The origin of the four stable Ribbon PFs (*h*) can be traced to PFs A11-12-13-1 of the A-tubule (*e*). The *yellow arrow* indicates the exact same anti-*Sp*Rib74/gold particle in *d* and *g*. Note that the shape of the partition-associated material appears to be altered somewhat in the extending Ribbon (*g* and *h*) from that in the intact A-tubule (*e*). *i–k*, models depicting the location of the partition material (*i* and *j*) and the same DMT→Ribbon transition in *k* as shown in *d* but with model superimposed over the EM structure, depicting the location of the stable Ribbon of PFs. *All panels, A*, A-tubule (*magenta*); *B*, B-tubule (*blue*); *MIP2* (*red*); *MIP3* (*yellow*); *IJ* protein (*purple*); *R*, Ribbons; *arrowheads*, immunogold particles; partition material, *stippled orange*. Cross-sections (*e–h*) and two-dimensional models (*i* and *j*) are viewed from the proximal (−) to the distal (+) end of the DMT→Ribbon transition; in longitudinal view (*d* and *k*) the proximal (−) end is toward the left, with polarity determined as in Fig. 7. *Scale bars, a–d* and *k*, 50 nm; *e–h*, 10 nm.

To localize the stable Ribbons, it was necessary to prepare reproducible samples of DMTs that transitioned at their ends into Ribbons. This was accomplished by extending the thermal fractionation beyond the melting of most of the B-tubules or by partial Sarkosyl extraction (Figs. 2*f*, 6*a*, and 7), whereby the A-tubules begin to disassemble, ultimately leaving stable Ribbons extending from the ends of remaining A-tubules. By interrupting this disassembly process, we obtained “DMT→Ribbon transitions” (Figs. 7 and 8), which we imaged by cryo-EM/ET. Analysis of tomograms of DMT→Ribbon transitions showed that the extending Ribbon still maintained its curved arc from the A-tubule in cross-sectional views and that the remaining A-tubule still possessed native structures (Figs. 7 and Fig. 8, *e–h*), *e.g.* microtubule-inner protein MIP2 (attached to the lumenal sides of PFs A9–10) and the inner A-B junction, including the IJ protein(s) and MIP3 (8, 11). The retention of these structures allowed us to identify with precision the specific Ribbon PFs in the DMT (see below).

To assist in localizing the stable PFs, it was advantageous first to localize *Sp*Rib74 and *Sp*Rib85.5. In our EM observations of both negatively stained and cryo-preserved DMT→Ribbon transitions, only a single Ribbon was seen to emerge from the A-tubule (Figs. 6*a* and 8, *a–d*), in agreement with the biochemical evidence for a single Ribbon per DMT (Fig. 5*c*). By immuno-negative staining EM, our anti-*Sp*Rib74 antibodies did not label intact DMTs or intact A-tubules and instead only labeled the extending Ribbons (Fig. 8*b*). Because *Sp*Rib74 and *Sp*Rib85.5 co-fractionate to the same compartment biochemically (Fig. 3*c*, *lane 6*) and co-localize to Ribbons by immuno-EM (Figs. 6*d* and 8, *b–d*), we continued the cryo-EM/ET analysis using only anti-*Sp*Rib74.

**FIGURE 9.**
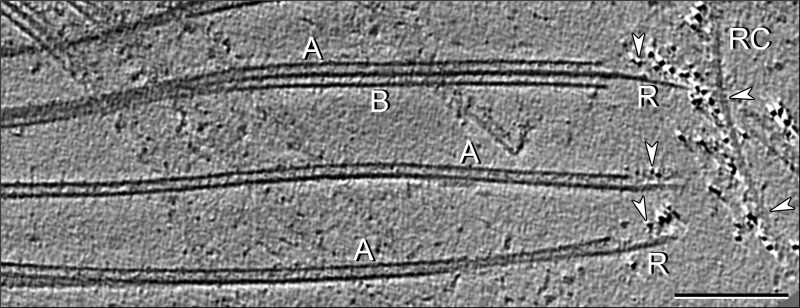
**Immuno-cryo-ET of DMT→Ribbon transitions.** Tomographic slice of DMT→Ribbon transitions after immunolabeling with anti-*Sp*Rib74/gold antibody. The gold-antibody complexes heavily label both the Ribbon controls (*RC*) and Ribbons (*R*) emerging from the A-tubules but only on the side facing the lumen of the A-tubule. *White arrowheads*, gold particles; *A*, A-tubules; B, *B*-tubules. *Scale bar*, 100 nm.

Next, we examined DMT→Ribbon transitions by a new hybrid technique, *i.e.* immuno-cryo-EM/ET (Figs. 8, *c–h*, and 9). We attached samples to thin, continuous carbon films covering EM grids, incubated the grids in anti-*Sp*Rib74 antibody, followed by gold-conjugated secondary antibody, and rapidly froze the grid specimen. For an internal control, we mixed purified Ribbons with the DMT→Ribbon transitions. By both immuno-cryo-EM and immuno-cryo-ET intact DMTs and A-tubules were not labeled, and anti-*Sp*Rib74/gold labeling occurred only along one side of the Ribbons projecting from A-tubules and along the purified control Ribbons. In fact, in both immuno-negatively stained and immuno-cryo-EM/ET samples, gold labeling only appeared along the concave side of the Ribbon facing the lumen of the A-tubule (Fig. 8, *b–k*). Because there is no evidence that *Sp*Rib74 (or *Sp*Rib85.5) forms PFs of MTs, we conclude that it interacts with or forms part of the partition material (Fig. 2, *a–d*, and later in this report). Because in secondary antibody labeling the gold particles can be up to 23 nm from the antigen and the gold position may bind at different angles to the antigen, the precise position and periodicity of the *Sp*Rib74 antigen could not be determined; however, the molar ratio (Fig. 5*b*) is consistent with Rib74 and Rib85.5 forming heterodimers that alternate along the Ribbon with a 16-nm periodicity.

The immuno-cryo-EM and three-dimensional immuno-cryo-ET data of DMT→Ribbon transitions also allowed us to determine the location of the stable Ribbon PFs within the DMT (Fig. 8, *c–k*). The intact A-tubule is easily identified by several structural markers as follows: (i) its prominent 16-nm repeating MIP2; (ii) the depolymerizing B-tubule (Figs. 2*f* and 7), which in cross-section occasionally appears as a hook-shaped structure consisting of PFs B9/10, MIP3, and the IJ protein(s); and (iii) the partition-associated material (Fig. 2, *a* and *c*). Typically, DMT→Ribbon transitions show four stable PFs emerging from the A-tubule, and by tracing these PFs back to the A-tubule, their identities were determined to be A11-12-13-1 (Fig. 8, *d–h*). We found it nearly impossible to obtain DMT→Ribbon transitions where the Ribbon is reduced to only three PFs, because if the thermal treatment is extended to eliminate the fourth PF, the integrity of the A-tubule is lost and the identity of the remaining three PFs cannot be determined without using the structural markers of the A-tubule as reference points. Despite this limitation, we found a few instances where a single PF persists after the other three PFs have terminated (see below).

##### Antibody Localization of Tektins

**FIGURE 10.**
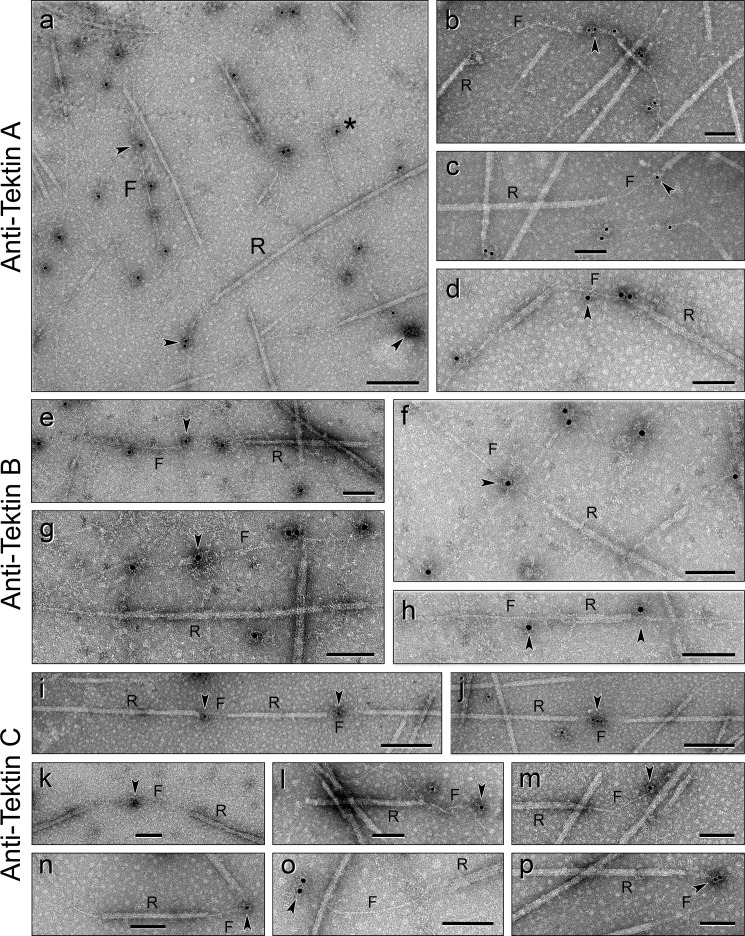
**Ribbon**→**filament transitions immunolabeled with anti-tektin antibodies.** The bare connecting or extending filaments are labeled by all three of the specific anti-tektin antibody/gold particles (marked by *arrowheads*), indicating that the single filament is composed of tektins A, B, and C; the statistics of the gold labeling are given in the text. *a*, nonspecific background labeling by *gold* is low (one of the few exceptions is labeled by an *asterisk*). Rarely do these antibodies/gold particles label Ribbons, indicating that the epitopes of tektins in the intact Ribbon are inaccessible to the antibodies. *a–d*, anti-tektin-A labeling of Ribbon→filament transitions. *a, S. purpuratus* Ribbons; *b–d*, *L. pictus* Ribbons. *e–h*, anti-tektin-B labeling of *S. purpuratus* Ribbon→filament transitions. *i–p*, anti-tektin-C labeling of *L. pictus* Ribbon→filament transitions. *Scale bars, a, i*, and *j*, 200 nm; *b–h* and *k–p*, 100 nm.

The method to prepare DMT→Ribbon transitions produced almost no examples with extending stable filaments before the structural integrity of the A-tubules was lost. Therefore, in the case of *S. purpuratus*, we first isolated Ribbons of three PFs and then partially extracted them with Sarkosyl-urea (at concentrations used to biochemically purify tektin filaments: Figs. 2*h* and 4*a, lane 3*) to produce Ribbon→filament transitions. These specimens had partially lost Ribbon PFs, leaving a single ∼5-nm wide filament either extending from the ends of Ribbons or connecting intact Ribbon segments (Figs. 6, *b* and *c*, and 10). In the case of *L. pictus*, we noted that these Ribbons naturally disassembled into Ribbon→filament transitions during storage on ice. In both cases, the extending filaments appeared to be similar, if not identical, to purified tektin filaments (Fig. 2*h*). This condition allowed us to examine the Ribbon and the extending or bare filament by antibody labeling.

To identify and localize all three tektins, we used three affinity-purified anti-tektin antibodies, individually specific for tektins A, B, or C (Fig. 4*a, lanes 7–9*) (previous work had used only a single antiserum against a mixture of all three tektins (32, 61)). Ribbon→filament transitions were separately incubated with anti-tektin A, B, or C, followed by gold-conjugated secondary goat anti-rabbit IgG. Nearly identical results were obtained with both species and with each antibody; the results are shown in Fig. 10. Whereas the intact Ribbons were rarely labeled with anti-tektins, the extending single filaments were frequently labeled with each of the anti-tektins, where the percent labeling of filaments (compared with Ribbons) was 91% for anti-tektin A (*n* = 189 gold particles bound to either filaments or to Ribbons), 99% for anti-tektin B (*n* = 171), and 85% for anti-tektin C (*n* = 208). Because all three anti-tektin antibodies are rabbit IgGs, double labeling was not possible. Nevertheless, because a single filament extends from a Ribbon and labels separately with each anti-tektin, these results indicate that the single filament contains all three tektins, *i.e.* that tektin C is not located at sites different from tektins A and B, as questioned earlier (27). Similar to previous investigations (32), the epitopes of tektins in the intact Ribbon structure are inaccessible to antibody or are masked by tubulin and/or other Ribbon proteins.

##### Antibody Localization of Tubulin

**FIGURE 11.**
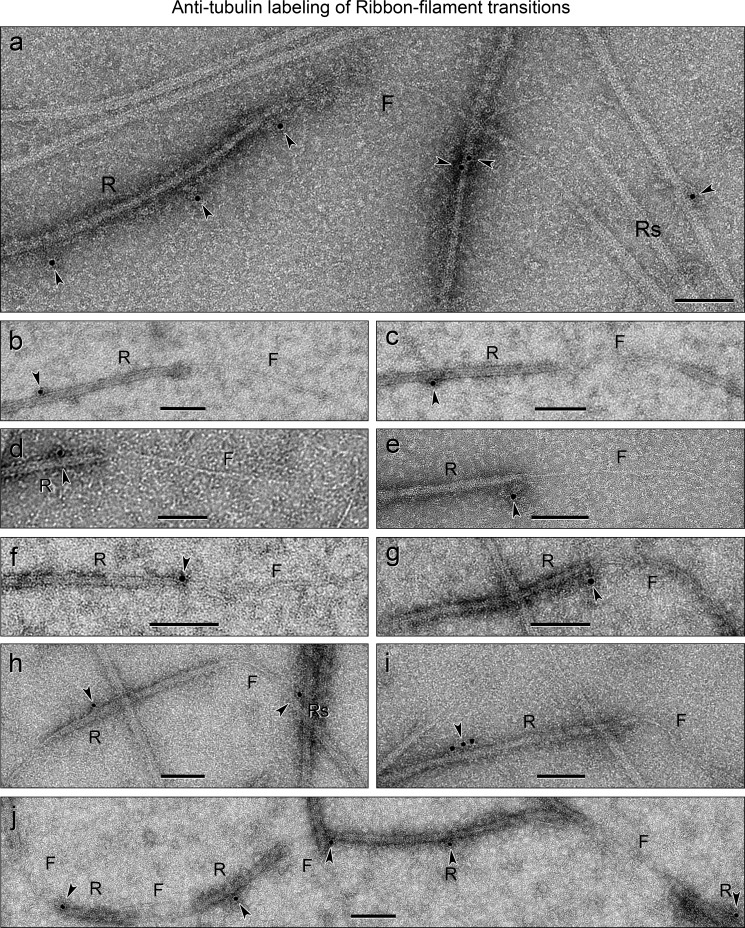
**Ribbon**→**filament transitions immunolabeled with anti-tubulin antibodies.** Only the Ribbons (*R*) containing tubulin protofilaments are immunolabeled (*arrowheads*); the bare or extending filaments (*F*) are rarely labeled with anti-tubulin. The statistics of the gold labeling are given in the text. *Scale bars, a–j*, 100 nm.

To test whether the bare and/or extending filament is a tubulin PF, perhaps stabilized by tektin fibrils, we also immunostained Ribbon→filament transitions with anti-tubulin antibodies (Fig. 11). We tried seven different commercial anti-tubulin (mostly polyclonal) antibodies, but the one that worked best was a mouse monoclonal antibody specific for acetylated α-tubulin (Fig. 4*a*, *lane 6*). Although the labeling density and nonspecific background were low with this antibody, the percentage of specific labeling of Ribbons compared with filaments was high, 97% (*n* = 133). These results were opposite those with anti-tektin antibodies, and thus the filament does not contain acetylated α-tubulin detectable by this procedure. This conclusion is supported by the evidence that biochemically isolated tektin filaments contain almost no detectable αβ-tubulin (Fig. 4*a, lane 3*).

##### Structure of the Isolated Ribbon and the Arrangement of Its Associated Proteins

**FIGURE 12.**
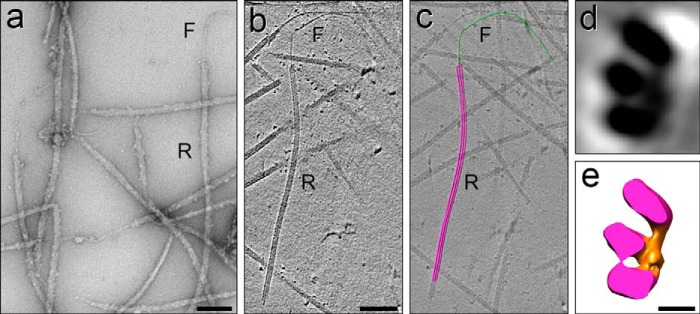
**EM of Ribbon-filament transitions.** *a–c*, Ribbon-filament transitions imaged by negative staining EM (*a*) and by cryo-ET (*b*) and modeled (*c*). In all cases, a single filament (as in Figs. 6, *b* and *c*, 10, and 11) appears to emerge from the middle of the Ribbon, as seen in two dimensions; however, the resolution in the z-direction of the ET data is not sufficient to locate the filament in/on the Ribbon in three dimensions unambiguously. *d* and *e*, cross-sectional view of a 24-nm thick slice through a subtomographic average of a Ribbon (*d*) and an isosurface rendering representation (*e*) reveals details of the partition-associated material (*orange*) asymmetrically bound to three Ribbon PFs (*magenta*); however, the structure of the partition material changes along its axis (see supplemental Movie S1). Comparison of these images with intact DMTs (Fig. 2, *a* and *c*) and immuno-cryo-ET images of DMT→Ribbon transitions (Fig. 8, *d–h*) localizes these three stable Ribbon PFs to either A11-12-13 or A12-13-1, with the ambiguity being due to the loss of polarity information during the preparation of the specimen. *Scale bars, a–c*, 100 nm; *d* and *e*, 5 nm.

**FIGURE 13.**
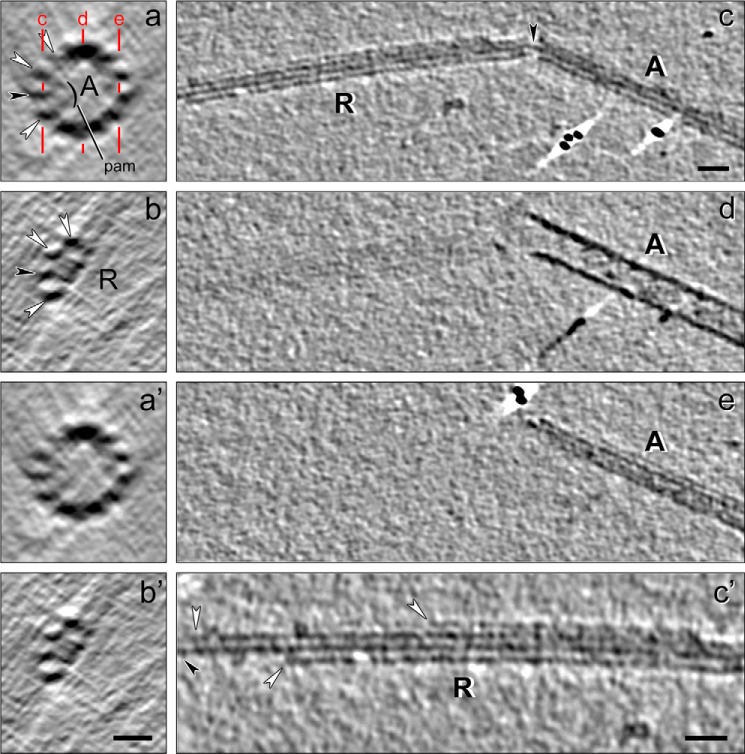
**Cryo-electron tomogram of an A-tubule**→**Ribbon**→**Protofilament transition, following extended thermal fractionation.** *a, a′, b*, and *b′*, A-tubule (*A*) (*a* and *a′*) has disassembled into a protruding Ribbon (R) (*b* and *b′*) of four PFs with accompanying partition-associated material (*pam, bracket*); tomographic slices (labeled and unlabeled) show cross-sectional views. Because the structural markers necessary to orient the A-tubule (*e.g.* MIP2 and the B-tubule hook, see Fig. 7) have been lost, the polarity of the A-tubule is ambiguous, and thus only a range of PFs is given for the PFs that appear to be in contact with the partition-associated material. In the displayed orientation the A-tubule→Ribbon transition matches best to the structural appearance of the partition-associated material in an A-tubule viewed from the proximal/minus to distal/plus end, as displayed in Figs. 2 and 7, supplemental Movie S1, and Ref. 59. Note, however, that the partition-associated material appears to change its shape on the Ribbon (*b* and *b′*) compared with that in the intact A-tubule (*a* and *a′*), preventing an unambiguous polarity determination. *c, d, e*, and *c′*, longitudinal views of the same A-tubule→Ribbon transition shown in *a–b′*; the tomogram of the A-tubule was sliced along the planes indicated by *red lines* in *a. Black arrowheads* in *a–c* point to the same PF, continuous through the *bend/break* in the Ribbon (*c*). The successive longitudinal slices (*c–e*) show the Ribbon (*R*) protruding from the A-tubule (*A*) (*c*) but absent in slices below this level (*d* and *e*). *c′*, high magnification view of the termination of the Ribbon, with the two peripheral PF (*white arrowheads*) ending first, and eventually a single most stable filament (*black arrowhead*), which corresponds to the continuous PF in the *bend/break* (*c*), extending the furthest. It is not yet known whether this last, most stable PF is a tubulin protofilament or a tektin filament. *Scale bars, a, a′, b*, and *b′*, 10 nm; *c–e*, 20 nm; *c′*, 20 nm.

In an effort to determine the arrangement of *Sp*Rib74, *Sp*Rib85.5, and the tektin filament associated with Ribbons, we imaged Ribbon→filament transitions purely by negative staining EM (Fig. 12*a*) and cryo-ET (Fig. 12, *b–e*) without immunolabeling. It is important to point out that Sarkosyl does not significantly alter the native-like protofilament structure of Ribbons at 3–4 nm resolution, because Ribbons retain the curvature of the A-tubule wall (Figs. 8, *g* and *h*, 12, *d* and *e*, and Fig. 13, *b* and *b′*), and they retain the 8-nm axial repeat of their tubulin dimers, which still bind kinesin (63); however, the shape of the partition-associated material in the protruding or isolated Ribbons seems to change somewhat compared with that in intact A-tubules (Figs. 8, *e–h*, and 12). In cryo-tomograms, the PFs of the Ribbon appear tightly joined by the partition material facing the lumen of the A-tubule (Fig. 12, *d* and *e*). The partition material has an asymmetric appearance across the Ribbon both in native DMTs and isolated Ribbons (compare Figs. 2, *a* and *c*, 12, *d* and *e*), with the largest mass situated over and along PFs A12/13, coinciding with the lumenal component (59) and MIP4 (11); nevertheless, the partition material makes contact with each of the four PFs (see supplemental Movie S1) with some electron density near PFs A1 and A11. A single filament, shown to be composed of tektins A, B, and C (Fig. 10), emerges from very near or at the position of the middle filament of the Ribbon (Fig. 12, *a–c*); however, the origin of the tektin filament in or on the Ribbon is not certain. The filament can be easily seen after it has emerged from the Ribbon in two-dimensional views (Figs. 6, *b* and *c*, and 12, *a–c*). The difficulty in locating the tektin filament precisely stems from the fact that, at the point where the filament emerges from the Ribbon, there is considerable noise (Fig. 6*c*). This noise may be due to interference from dissociating (denatured) tubulin, *Sp*Rib74, and *Sp*Rib85.5, some of which remains adhered to the tektin filament. However, we do occasionally observe a single stable protofilament of the Ribbon remaining after extended thermal fractionation (Fig. 13), but after such extended treatment, the positional markers required to orient the Ribbon are not unambiguously identifiable.

##### Summary of Findings

1) Sarkosyl-stable Ribbons from echinoderm flagella contain acetylated α-tubulin, β-tubulin, *Sp*Rib45, and >95% of tektins ABC, and >95% of *Sp*Rib74/*Sp*Rib85.5, *i.e.* these proteins are not detectable in other compartments of the DMT. 2) Sarkosyl-purified Ribbons consist of three PFs in sea urchin *S. purpuratus* (Fig. 2*g*), four PFs in sea urchin *L. pictus* (63), and three PFs in *Chlamydomonas* (12); there is only one tektin-/Rib74-/Rib85.5-containing Ribbon per DMT. 3) DMT→Ribbon transitions from *S. purpuratus* prepared for EM contain a single Ribbon of four PFs, corresponding to PFs A11-12-13-1 of the A-tubule. Of these, the most stable three PFs have yet to be identified unambiguously, but they include two to three of the “partition” PFs originally suggested (12), *i.e.* PFs A12-13-1 or A11-12-13. 4) Each Ribbon contains a single hyper-stable tektin filament, which is ∼5 nm wide and smooth, is composed almost exclusively of tektin A, B, and C in equimolar amounts, and does not contain detectable levels of tubulin. 5) *Sp*Rib74 (and probably *Sp*Rib85.5) is associated with or forms the material located on the lumenal side of the partition. 6) Sea urchin Ribbon protein *Sp*Rib45 is a homologue of *Chlamydomonas Cr*Rib43a, now completing the list of major structural proteins of the Ribbon, conserved from protists to human. 7) New methods were developed, *i.e.* immuno-cryo-EM, immuno-cryo-ET, and the isolation of DMT→Ribbon and Ribbon→filament transitions, which will be useful for future studies.

## DISCUSSION

### 

#### 

##### Tektin Filament Is an Integral Polymer of the Ribbon

We have determined the composition and stoichiometry of tektins in the intact polymer and narrowed their location to the region of PFs A11-12-13-1. The possibility cannot be excluded that three separate tektin coiled-coil fibrils (two 2AB fibrils and one CC fibril) are in parallel contact with the 3–4 PFs of the Ribbon, where the apparent 5-nm wide filament (Fig. 2*h*) would be an artifact of the winding together of the fibrils following urea extraction. However, in such a case the individual fibrils on the surface of the Ribbon might be expected to stain with antibodies, but they do not (Fig. 10). Furthermore, it is unlikely that such an artifactual winding would produce micron-long filaments of constant diameter (Fig. 2*i*). Alternatively, because tektin and IF proteins share many properties (27, 30, 79), tektin filaments may be super-coiled like IFs, which have been shown to have a super-coiled structure with a 96-nm helical supertwist (80). This IF structure-resembling model was previously proposed for tektin filaments (44) and would be consistent with our data as follows: a 5-nm diameter filament with a 96-nm helical pitch (the evolutionarily conserved, fundamental repeat of all axonemes), consisting of a core filament of two coiled-coil tektin AB heterodimers, and a less stable coiled-coil fibril of tektin CC homodimers polymerized around this core filament. Moreover, the tektin filament appears smooth both in negative stain (Figs. 2, *h* and *i*, 6, *b* and *c*, and 12*a*) and by cryo-EM/ET (Fig. 12*b*), without hypothesized side projections (27, 62).

Although the position of the single tektin filament coincides in two-dimensional projections with the middle PF of the Ribbon (Figs. 6, *b* and *c*, and 12, *a–c*), by three-dimensional cryo-ET analysis it is currently impossible (as explained under “Results”) to follow the ∼5-nm diameter filament back to its exact origin in the Ribbon, where it either replaces a tubulin-PF or lies along the surface of the central tubulin PF. In *Chlamydomonas*, it has been reported that >50% of the single tektin isoform is solubilized by 0.5% Sarkosyl extraction of axonemes after 1 h (25). In this case, at least in *Chlamydomonas*, tektin cannot form one of the Ribbon PFs, leaving two possibilities for the location of the tektin filament.

The first possibility is that in all species the tektin filament may be located within the partition-associated material. In cross-sectional cryo-ET slices of sea urchin Ribbons, the continuous density occurring in the partition material along PF A12/A13 might correspond to a continuous tektin filament (Fig. 12, *d* and *e*, and supplemental Movie S1). If so, the tektin(s) must then be extractable from *Chlamydomonas* Rib72-containing Ribbons without disrupting their 3-PF structure. These considerations beg the question of whether or how tektin, *Sp*Rib75 (*Cr*Rib72), *Sp*Rib85.5, and *Sp*Rib45 might interact with each other and/or with tubulin to stabilize the Ribbon PFs.

The second possibility argues against the complete evolutionary conservation of DMT structure. In this model with the evolution of two new tektins (from the single tektin in *Chlamydomonas* to three tektins in metazoans), the more robust metazoan tektin filament (with a tektin AB-core surrounded by tektin C, forming an ∼5-nm diameter filament) may have evolved to mimic a tubulin PF inserted into the Ribbon, *e.g.* as the middle PF (see Fig. 13) (44, 63). This model will be proven or disproven by further structural studies.

##### What Are the Functions of Ribbon Proteins?

Ribbon proteins may be expected to function in ciliary/flagellar assembly, stability, motility, and/or signaling and other MT systems.

##### Centriole and Cilia Assembly

The role of tektins in ciliogenesis and MT turnover has been rigorously investigated (35, 36, 51), and tektins, Rib*Sp*74, and Rib*Sp*85.5 are present in centrioles and basal bodies, the templates for DMTs (21, 26, 37). Thus, these Ribbon proteins might be expected to be positioned in triplet MTs in the same locations as they are in DMTs. However, in cryo-ET studies of *Chlamydomonas* and *Trichonympha* basal bodies (6, 7), the partition material is barely evident, if at all present, compared with that in *Chlamydomonas* and sea urchin DMTs (compare with Fig. 2, *a–d* and Ref. 11).

During their assembly, axonemal microtubules must configure numerous axial repeats that are multiples of the 8-nm tubulin-dimer repeat and subdivisions of the fundamental 96-nm axonemal repeat, *e.g.* the 8-nm spacing of MIP1; the 16-nm spacing of MIP3; the 16/48-nm spacing of MIP2; the alternating 24/32/40-nm spacings of radial spoke triplets; the complex spacings of the inner dynein arms; the 24-nm spacing of outer dynein arms, and the 96-nm repeat of the nexin-dynein regulatory complex (65). It is not clear how these periodicities are established. Tektin subunits and/or tektin filaments have observed spacings of 4, 8, 16, and 48 nm (30, 34, 61, 63). Therefore, the tektin filament is currently the only known candidate with the properties to act potentially as a primary molecular ruler that specifies most observed axonemal periodicities.

##### DMT Stability

The unusual stability of DMTs (81) may be dependent on the hyperstability of the Ribbon regulated by cooperative interactions of all of the major proteins, *i.e. Sp*Rib45 (*Cr*Rib43a), *Sp*Rib74 (*Cr*Rib72), *Sp*Rib85.5, tektins, and acetylated α-tubulin, with the latter being associated with stable MTs in general (72). The site of acetylation, Lys-40 (82), faces the MT lumen (83), and in DMT Ribbons the lumenal face appears to be covered significantly by the partition material. This arrangement could explain why immunogold labeling of Ribbons with anti-acetylated α-tubulin (Fig. 11) is infrequent but highly specific, as if Rib75, Rib85.5, and/or tektins are masking most of the acetylated sites (and perhaps interacting with them). It should be noted that the *Mm*EFHC1 (homologue of *Cr*Rib72 and *Sp*Rib74) is reported to bind to α-tubulin (23) and is associated with tektin-containing cytokinesis midbody MTs, which are as stable as ciliary A-tubules (38). In terms of tektins, they have several structural similarities to IF proteins (mentioned above) and are reported to be phosphoproteins (84). Thus, the assembly of tektin filaments and their subsequent hyper-stability may be coupled to a dephosphorylation of soluble phosphorylated subunits, analogous to nuclear lamins (85).

##### Motility and Signaling

*Cr*Rib72, *Sp*Rib74, and *Sp*Rib85.5 possess three DM10 domains of unknown function and two EF-hand motifs (19, 47) predicted to bind Ca^2+^ ions (86). Furthermore, the sensitivity of *Cr*Rib72 to trypsin digestion is affected by Ca^2+^ concentration (19), suggesting that the conformation of Rib72 homologues is altered by Ca^2+^. Of likely relevance, the switch from ciliary to flagellar waveform in *Chlamydomonas* and ciliary reversal in other organisms occur at 10^−6^ m Ca^2+^ (87–90), and there are several Ca^2+^-binding complexes in axonemes, including the central pair MT complex and the calmodulin- and radial spoke-associated complex (91), which could contribute to regulating motility. Potentially, Ca^2+^-induced conformational changes of Rib72 homologues might alter the twist or persistence length of DMTs (92), effectively regulating bending and waveform. Such a Ribbon Ca^2+^-regulation system seems to be absent in sensory cilia, because in *Caenorhabditis elegans*, which has only nonmotile sensory cilia, the *Ce*Rib72 homologue lacks an EF-hand motif. Thus, Rib72 homologues presumably function in two ways, first as Ca^2+^-dependent motility regulators, and second, in an as yet undefined way in both motile and nonmotile cilia, if only for the assembly and stability of DMTs discussed above.

Given the association of the tektin filament with the partition region extending toward the inner A-B junction, the interaction of tektins with dynein is peculiar and intriguing. The first reports of an association between tektin and dynein heavy chains (43) and between tektin and nexin (42) were the basis for suggesting that tektins were located closer to PFs A1–3 (44). The finding that the dynein regulatory complex (DRC) was actually a Nexin-DRC complex that is in direct contact with inner arm dyneins (9, 41) seemed to explain the earlier tektin-dynein/nexin findings. In addition, an 80% reduction in *Chlamydomonas* tektin was reported for the *n-drc* mutants, *ida6* and *pf3*, that lack inner dynein arm polypeptide “e” (25). More directly, Tanaka *et al.* (39) deleted Tektin-t in mice and showed a loss of inner dynein arms and an accompanying immotile cilia phenotype affecting sperm flagella and tracheal cilia. These studies raise fascinating questions of how the Nexin-DRC interacts with tektin(s) that are spatially separated.

The findings presented here should encourage and inform future approaches to analyzing the functions of stable ciliary protofilament Ribbons and their constituent proteins.

## Supplementary Material

Supplemental Data

## Footnotes

4 The abbreviations used are:

DMT: doublet microtubule
EFHC: EF-hand(s) in the carboxy terminus
ET: electron tomography
IF: intermediate filament
IEF: isoelectric focusing
*Lp*: *L. pictus*
MT: microtubule
PF: protofilament
Rib: Ribbon protein and proteins associated with isolated Ribbons
*Ce*: *Caenorhabditis elegans*
*Sp*: *S. purpuratus*
*Mm*: *M. musculus*
*Cr*: *C. reinhardtii*
DRC: dynein regulatory complex.
