# Supplementary material for: Insights into the Structure and Function of Ciliary and Flagellar Doublet Microtubules: TEKTINS, Ca2+-BINDING PROTEINS, AND STABLE PROTOFILAMENTS
Source: J Biol Chem. 2014 May 2;289(25):17427–44. doi: 10.1074/jbc.M114.568949 (PMC4067180; doi:10.1074/jbc.M114.568949)
Supplement: Supplemental Data [file supp_289_25_17427__index.html]

Insights into the structure and function of ciliary and flagellar doublet microtubules: tektins, Ca2+-binding proteins and stable protofilaments — Insights into the Structure and Function of Ciliary and Flagellar Doublet Microtubules — Ciliary Doublet Microtubules — Supplemental Data 

# Insights into the Structure and Function of Ciliary and Flagellar Doublet Microtubules

## Supplemental Data

**Files in this Data Supplement:**

- Supplemental movie S1 (.mov, 3.8 MB) - Movie S1: Cross-sectional tomographic view and model of a DMT. The movie traverses in 1-nm steps along 120 nm of an averaged DMT (*S. purpuratus*; compare with Fig. 2a-d and Fig. 7) viewed from the proximal (-) end to the distal (+) end. Certain densities appear to be continuous along the axis, while other densities appear as axially periodic globular structures. A, A-tubule (magenta numbered); B, B-tubule (blue numbered); MIP1 (light blue); MIP2 (red); MIP3 (yellow).
